# A Comparative Evaluation of Strain and Shear Wave Ultrasound Elastography for Characterizing Cervical Lymphadenopathy

**DOI:** 10.7759/cureus.100541

**Published:** 2025-12-31

**Authors:** Anil Rawat, Siddharth Mishra, Nitin Arun Dikshit, Priyanka Yadav, Saurabh Kumar, Himanshu Nirwal

**Affiliations:** 1 Department of Radiodiagnosis, King George's Medical University, Lucknow, IND; 2 Department of Radiology, King George's Medical University, Lucknow, IND

**Keywords:** cervical lymphadenopathy, diagnostic accuracy, shear wave elastography, strain elastography, ultrasound imaging

## Abstract

Background: Cervical lymphadenopathy has varied causes, from benign infections to malignancy. Accurate differentiation is crucial for staging and treatment. While fine needle aspiration cytology and histopathology are considered the gold standards, they are invasive procedures. Conventional ultrasound offers structural and vascular assessment but limited specificity. Elastography, including strain elastography and shear wave elastography (SWE), measures tissue stiffness, which is typically higher in malignancies.

Aim: This study aimed to assess the role of strain and SWE in differentiating benign from malignant cervical lymph nodes.

Methods: In this cross-sectional study at a tertiary care center, 97 cervical lymph nodes from patients aged 5-60 years were evaluated with B-mode ultrasound, color Doppler, strain ratio, and SWE. Histopathology/cytology served as the reference. Receiver operating characteristic analysis determined diagnostic performance.

Results: Malignancy was associated with older age, female sex, levels II and IV, absent hilum, heterogeneous echotexture, and peripheral vascularity (t = -3.64, p = 0.001). Malignant nodes had higher mean elasticity (72.06 vs. 9.23 kPa) and strain ratio (2.30 vs. 1.18) (t = -3.64, p = 0.001). SWE velocity cutoff 2.195 m/s achieved 100% sensitivity and 85% specificity (area under the curve, AUC = 0.940). A strain ratio cutoff of 1.465 achieved 91.5% sensitivity and 99% specificity (AUC = 0.956).

Conclusion: Strain and SWE, combined with conventional US, provide high accuracy in differentiating cervical lymph node pathology, thereby reducing the need for unnecessary invasive procedures.

## Introduction

Cervical lymphadenopathy, the enlargement of cervical lymph nodes, arises from diverse causes, including benign infections, lymphoma, and metastatic malignancies. Accurate evaluation is essential, particularly in oncology, as lymph node status influences staging, prognosis, and treatment [1-4]. While fine needle aspiration cytology (FNAC) and histopathology are considered the diagnostic gold standards, they are invasive, costly, and uncomfortable procedures. B-mode US, often complemented by color Doppler, is the primary imaging tool for assessing nodal morphology, echotexture, and vascularity. However, no US criterion reliably differentiates benign from malignant nodes, leading to frequent reliance on invasive confirmation [5,6].

US elastography addresses these limitations by assessing tissue stiffness, with malignancies typically exhibiting higher stiffness due to increased cellular density and fibrosis [7,8]. Two main techniques exist: strain elastography and shear wave elastography (SWE). Strain elastography utilizes manual compression to create elastograms, visually mapping stiffness and providing a strain index (SI), where a higher SI suggests malignancy [5,9,10]. Color-coded elastograms (blue for stiff, red/green for soft) aid visual differentiation, particularly when combined with conventional US. SWE, using acoustic radiation force impulses, quantifies stiffness in kilopascals (kPa) or shear wave velocity (m/s) [11,12]. It is automated, less operator-dependent, and more reproducible, enabling standardized cross-study comparisons. Elastography has been widely applied not only to lymph nodes but also to various other tissues and organs. Indeed, elastography has been widely applied not only to lymph nodes but also to the assessment of tendon pathologies [13], liver diseases [14], peripheral nerve evaluation [15], and pancreatic tissue [16], with its effectiveness repeatedly demonstrated in the literature.

Elastography enhances conventional ultrasound by improving differentiation between benign and malignant nodes, guiding FNAC/biopsy to the most suspicious targets, and potentially monitoring treatment response [12]. In prior studies, strain elastography and SWE have demonstrated high sensitivity and specificity, with SI and shear wave velocity as key discriminators. Limitations include operator dependence in strain elastography, SWE artifacts, equipment requirements, and the absence of standardized stiffness cutoff values [10,12].

Given these advantages and constraints, this study aimed to evaluate the role of strain and SWE parameters in differentiating benign from malignant cervical lymph nodes, thereby enhancing noninvasive diagnostic precision and reducing the need for unnecessary invasive procedures.

## Materials and methods

Study design and setting

This cross-sectional study was conducted in the Department of Radiodiagnosis at a tertiary care center in Northern India over a one-year period. The research was conducted in a clinical setting equipped with advanced US and elastography facilities, allowing for a comprehensive evaluation of patients with cervical lymphadenopathy.

Study participants

This study included patients aged 5-60 years with clinically palpable cervical lymph nodes or imaging-detected enlargement (as detected by CT, MRI, or sonoelastography) who provided written informed consent. Ninety-seven nodes were evaluated, including submental, submandibular, internal jugular (upper, middle, and lower), posterior triangle, and supraclavicular groups. Exclusion criteria were ongoing treatment for infectious/autoimmune conditions, post-chemotherapy or radiotherapy status, recent biopsy (within 15 days), and refusal to participate. Ethical approval was obtained, and informed consent was secured from all participants.

Sample size

The sample size was calculated based on a reported elastography sensitivity of 0.839 and a cervical lymph node prevalence of 0.44 [17], with a precision of ±0.11 and a 95% confidence level, yielding a requirement of 97 cases. Therefore, the final sample size was 97.

Data collection

Written informed consent was obtained, and patient details were recorded using a predesigned proforma. US examinations were performed using a Philips Affiniti 70G machine (Royal Philips N.V., Amsterdam, The Netherlands). Patients were positioned supine with their necks extended. B-mode ultrasound was used to assess lymph node size, shape (long-to-short-axis ratio), hilum status, vascularity, and cortical thickness, followed by strain and SWE.

SWE was performed using a 1-5 MHz curvilinear transducer, with the region of interest placed over the node parallel to the skin. Elasticity (kPa) and shear wave velocity (m/s) were recorded in triplicate and averaged. Strain elastography was done with a 5-12 MHz linear transducer using gentle compression, generating color-coded stiffness maps scored on a four-point scale [12]. The strain ratio was calculated by comparing the node with the adjacent neck muscle. Histopathology/cytology served as the reference standard; clinical treatment response determined benignity in nonbiopsied cases.

Statistical analysis

Statistical analysis was performed using Statistical Package for the Social Sciences version 24.0 (IBM Corp., Armonk, NY). Continuous variables were expressed as mean ± standard deviation, while categorical data were presented as percentages. The chi-square test was utilized for analyzing categorical variables, and the independent t-test was applied to compare continuous variables between groups. Multivariate analysis was conducted to determine independent predictors. Receiver operating characteristic (ROC) curve analysis was employed to evaluate the diagnostic performance of elastography parameters, with the area under the curve (AUC) calculated. A p value of less than 0.05 was considered statistically significant. Additionally, sensitivity, specificity, positive predictive value (PPV), and negative predictive value (NPV) were calculated to further assess diagnostic accuracy.

## Results

A total of 97 cervical lymph nodes were assessed for correlations with demographic, anatomical, and ultrasonographic factors in relation to diagnosis. Benign nodes were more common in the 10-30 years group (64.2%), while malignancies predominated in the 30-50 years (40.4%) and >50 years (27.7%) groups (χ² = 13.82, p = 0.003). Male patients had more benign (62.3%) and female patients more malignant (59.6%) nodes (χ² = 4.76, p = 0.029). Level I nodes were mainly benign (45.3%), whereas level II nodes were often malignant (36.2%) (χ² = 11.38, p = 0.010) (Table 1).

**Table 1:** Association of age, sex, and lymph node location with final diagnosis of benign vs. malignant cervical lymphadenopathy

Association of age, sex, and lymph node location with final diagnosis of benign vs. malignant cervical lymphadenopathy		Final diagnosis		p value
		Benign	Malignant	
Age (years)	<10	2 (3.8%)	0 (0.0%)	0.003
	10-30	34 (64.2%)	15 (31.9%)	
	30-50	11 (20.8%)	19 (40.4%)	
	>50	6 (11.3%)	13 (27.7%)	
Sex	Males	33 (62.3%)	19 (40.4%)	0.029
	Females	20 (37.7%)	28 (59.6%)	
Lymph node locations	I	24 (45.3%)	17 (36.2%)	0.010
	II	10 (18.9%)	17 (36.2%)	
	III	7 (13.2%)	5 (10.6%)	
	IV	2 (3.8%)	7 (14.9%)	
	V	10 (18.9%)	1 (2.1%)	

B-mode US showed a central fatty hilum in most benign (64.2%) but absent in malignant (80.9%) nodes (t = -13.54, p = 0.001). Benign nodes more often had normal echotexture (64.2%), while malignant nodes showed heterogeneity (66.0%) (t = -11.40, p = 0.001). Peripheral vascularity was predominantly malignant (87.2%), and malignant nodes were larger in both short- and long-axis diameters (t = -13.55, p = 0.001). Diameter ratio differences were insignificant (t = -1.13, p = 0.258) (Table 2).

**Table 2:** Ultrasonographic features differentiating benign and malignant cervical lymphadenopathy USG: ultrasonography

USG findings		Final diagnosis		p value
		Benign	Malignant	
Hilum appearance	Present	34 (64.2%)	9 (19.1%)	0.001
	Absent	19 (35.8%)	38 (80.9%)	
Echotexture	Normal	34 (64.2%)	2 (4.3%)	0.001
	Heterogeneous	6 (11.3%)	31 (66.0%)	
	Homogenous	13 (24.5%)	14 (29.8%)	
Vascularity	Normal	25 (47.2%)	0 (0.0%)	0.001
	Central	28 (52.8%)	6 (12.8%)	
	Peripheral	0 (0.0%)	41 (87.2%)	
Lymph node size (mm)	Short axis	10.66 ± 0.586	13.40 ± 1.17	0.001
	Long axis	12.94 ± 1.26	17.02 ± 1.66	
Diameter ratio (long/short axis)		1.23 ± 0.162	1.26 ± 0.115	0.258

**Figure 1:**
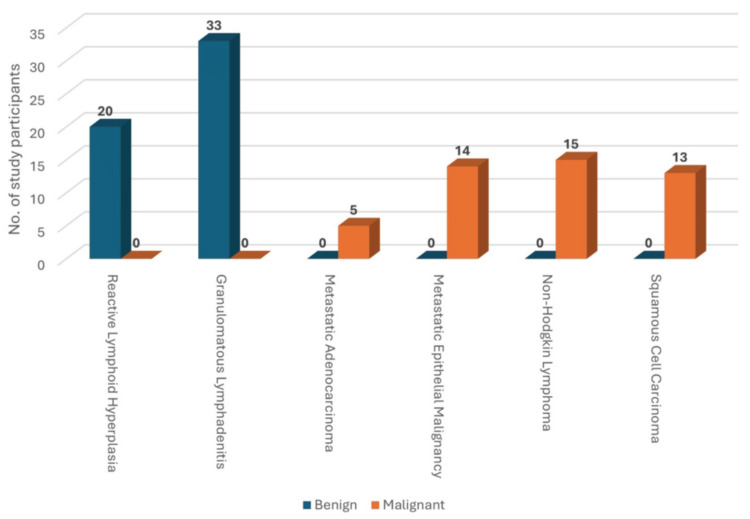
FNAC/histopathology findings in benign and malignant lymph nodes among the study participants FNAC: fine needle aspiration cytology

Granulomatous lymphadenitis (n = 33) and reactive lymphoid hyperplasia (n = 20) were exclusive to benign cases. In contrast, metastatic epithelial malignancy (n = 14), non-Hodgkin lymphoma (n = 15), and squamous cell carcinoma (n = 13) were exclusive to malignant cases (Figure 1).

Elastography revealed higher mean wave elasticity (72.06 ± 33.62 kPa), shear wave velocity (72.02 ± 33.55 kPa), and strain ratio (2.30 ± 0.667) in malignant nodes versus benign (1.18 ± 0.236) median wave velocity (9.23 ± 3.03 kPa and median shear wave velocity 9.29 ± 3.10 kPa, respectively) (t = -3.64, p = 0.001) (Table 3).

**Table 3:** Shear wave velocity measurements in benign and malignant lymph nodes among the study participants

Shear wave velocity (kPa)	Final diagnosis		t value	p value
	Benign	Malignant		
Mean wave elasticity	9.23 ± 3.03	72.06 ± 33.62	-13.54	0.001
Strain ratio	1.18 ± 0.236	2.30 ± 0.667	-11.40	0.001
Median shear wave velocity	9.29 ± 3.10	72.02 ± 33.55	-13.55	0.001
Mean shear wave velocity	2.05 ± 1.90	3.87 ± 3.02	-3.64	0.001

**Figure 2:**
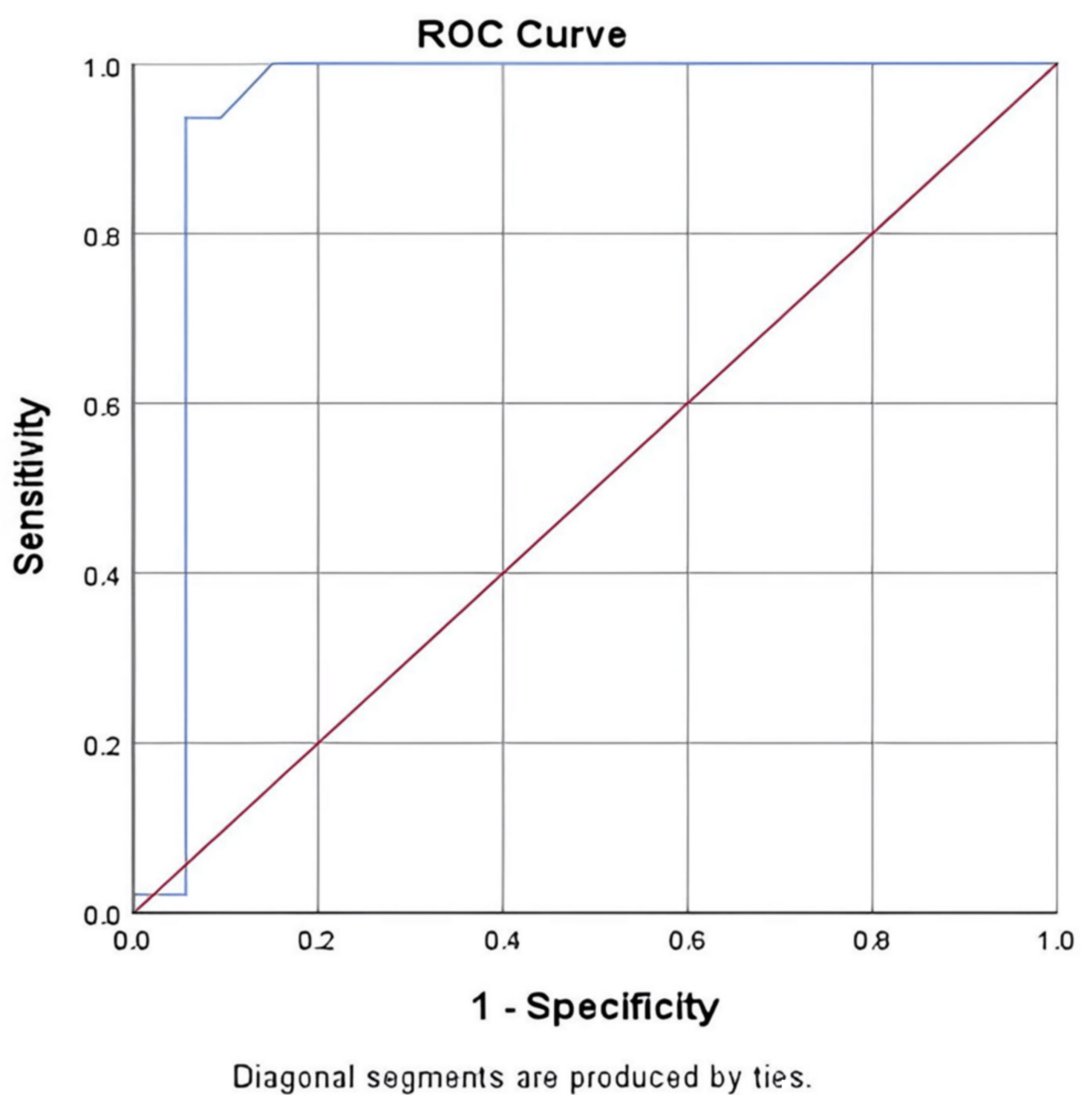
ROC of mean shear wave velocity for malignant and benign lymph nodes ROC: receiver operating characteristic

ROC analysis showed mean shear wave velocity AUC of 0.940 at 2.195 m/s cutoff, with 100% sensitivity, 85% specificity, and 92.5% accuracy (p = 0.001) (Figure 2, Table 4).

**Table 4:** ROC of mean shear wave velocity for malignant and benign lymph nodes parameter AUC: area under the curve; CI: confidence interval; PPV: positive predictive value; NPV: negative predictive value; ROC: receiver operating characteristic

Parameter	Value
AUC	0.940
Cutoff	2.195
p value	0.001
95% CI	0.879-1.000
Sensitivity	100%
Specificity	85%
PPV	86.9%
NPV	100%
Diagnostic accuracy	92.5%

**Figure 3:**
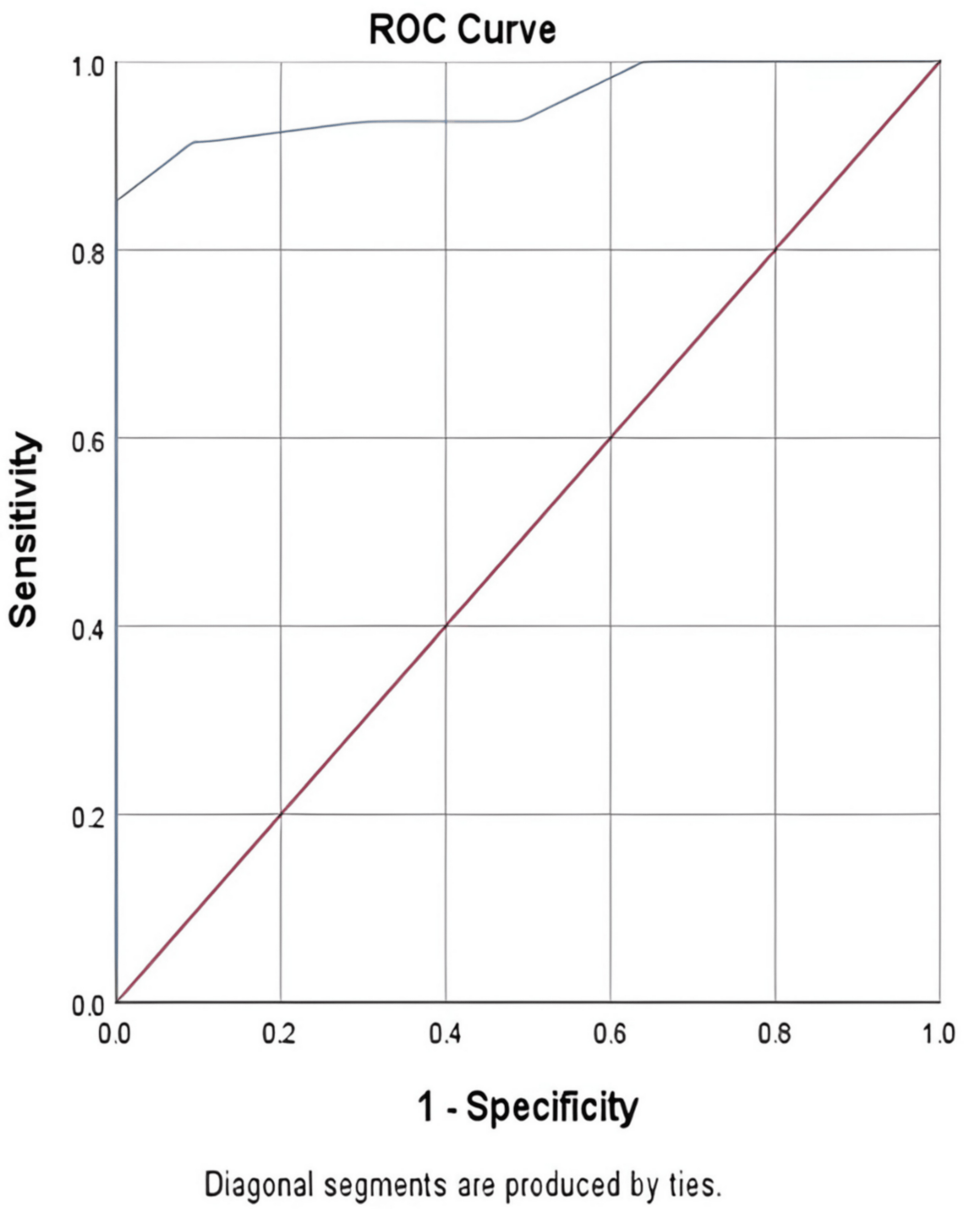
ROC of strain ratio for malignant and benign lymph nodes ROC: receiver operating characteristic

Strain ratio had an AUC of 0.956 at a 1.465 cutoff, with 91.5% sensitivity, 99% specificity, and 95.3% accuracy (p = 0.001) (Figure 3, Table 5).

**Table 5:** ROC of strain ratio for malignant and benign lymph nodes AUC: area under the curve; CI: confidence interval; PPV: positive predictive value; NPV: negative predictive value; ROC: receiver operating characteristic

Parameter	Value
AUC	0.956
Cutoff	1.465
p value	0.001
95% CI	0.914-0998
Sensitivity	91.5%
Specificity	99%
PPV	98.9%
NPV	92.1%
Diagnostic accuracy	95.3%

## Discussion

The primary aim of this study was to evaluate the role of strain and SWE in differentiating benign from malignant cervical lymph nodes. Our results confirm that both techniques provide excellent diagnostic performance, offering quantitative and reproducible measurements of tissue stiffness that significantly enhance the discriminatory capability of conventional ultrasound [18].

Malignant nodes in our cohort exhibited markedly higher stiffness than benign nodes. Mean SWE in malignant nodes was 72.06 ± 33.62 kPa compared to 9.23 ± 3.03 kPa in benign nodes. Similarly, the mean strain ratio was 2.30 ± 0.667 in malignant nodes vs. 1.18 ± 0.236 in benign (p = 0.001 for both). These findings align with the well-documented principle that malignant tissues exhibit higher stiffness due to increased cellular density, fibrosis, and alterations in the extracellular matrix.

Our results are consistent with prior studies. Mattoo et al. [19] found significantly higher shear wave velocities in malignant nodes (6.89 ± 2.70 m/s) compared to benign (2.04 ± 0.44 m/s), while Chavoshi et al. [20] reported values of 3.56 ± 0.7 m/s for malignant and 2.44 ± 0.3 m/s for benign nodes. Gupta et al. [21] demonstrated that strain ratios ≥2 were seen in most malignant (20/21) and <2 in most benign (27/29) nodes, reinforcing its diagnostic utility.

ROC analysis in our study showed that the mean shear wave velocity achieved an AUC of 0.940 (95% CI = 0.879-1.000) with a 2.195 m/s cutoff, yielding 100% sensitivity, 85% specificity, 86.9% PPV, and 100% NPV. All malignant cases were correctly identified, with no false negatives, making this parameter particularly valuable in clinical settings where missing malignancy is unacceptable. Chavoshi et al. [20] reported a slightly higher optimal cutoff (2.71 m/s; AUC = 0.92, sensitivity = 82.7%, specificity = 84.2%), and Mattoo et al. [19] found a cutoff of 2.8 m/s (sensitivity = 94.8%, specificity = 97.6%). The slightly lower cutoff in our study likely reflects population, anatomical, and equipment differences, but it increased sensitivity without markedly reducing specificity, making it well-suited for early detection.

The high diagnostic capability of shear wave velocity is further supported by Gao et al. [22], whose meta-analysis of 16 studies (>1,400 lymph nodes) found pooled AUCs above 0.88 for SWE parameters, and by Sasaki and Ogura [23], who reported malignant nodes with stiffness values averaging 105.9 ± 5.2 kPa, consistent with our observation of markedly elevated elasticity in malignancy.

Strain ratio analysis showed similarly robust results, with an AUC of 0.956 (95% CI = 0.914-0.998) at a 1.465 cutoff, yielding 91.5% sensitivity, 99% specificity, 98.9% PPV, and 92.1% NPV. These values are comparable to those reported by Vineela et al. [24], who used a cutoff of 2.0 (93% sensitivity, 96% specificity), and Gupta et al. [25], who reported 95.24% sensitivity and 93.10% specificity at the same threshold. Moharram et al. [26] found mean strain ratios of 3.4 ± 1.2 in malignant vs. 1.2 ± 0.3 in benign nodes, while Seetharam et al. [27] achieved 92% overall accuracy with a cutoff near 2.0.

Our study's slightly lower strain-ratio cutoff (1.465) may help detect malignancies at earlier stages, when stiffness is elevated but below conventional thresholds. Maintaining 99% specificity and high sensitivity minimizes false positives while avoiding missed malignancies, reducing unnecessary biopsies, and ensuring timely diagnosis.

Compared with individual B-mode and Doppler parameters, elastography provided superior accuracy. While absent hilum, heterogeneous echotexture, and peripheral vascularity were each strongly associated with malignancy (p = 0.001), their diagnostic performance was lower than that of elastography. Peripheral vascularity was exclusive to malignant nodes in our cohort, in agreement with Gupta et al. [25], Moharram et al. [26], and Seetharam et al. [27], but the addition of stiffness quantification further improved diagnostic confidence.

Integrating strain and SWE with conventional ultrasound creates a comprehensive evaluation strategy. B-mode provides morphological assessment-shape, echotexture, hilum status, and size-while color Doppler supplies vascular patterns. Elastography adds biomechanical data, quantifying stiffness objectively. This multimodal approach reduces reliance on a single feature, addressing the overlap in appearances between benign and malignant nodes that can limit conventional ultrasound.

This study shows that strain and SWE reliably distinguish benign from malignant cervical lymph nodes. Cutoffs of 2.195 m/s (shear wave velocity) and 1.465 (strain ratio) achieved excellent sensitivity and specificity, enhancing accuracy when combined with conventional ultrasound and Doppler, and reducing unnecessary invasive procedures. Findings align with Mattoo et al. [19], Chavoshi et al. [20], Gupta et al. [21], Vineela et al. [24], and Moharram et al. [26].

The study has a few limitations to consider when interpreting the results. The sample size was relatively small, with fewer benign cases than malignant ones, potentially limiting statistical power. Being a single-center study, the findings may not be fully generalizable to other populations or clinical settings. US and elastography are inherently operator-dependent, which could introduce variability despite standardization of techniques. There is also the possibility of overlap in stiffness patterns, as reactive or granulomatous lymph nodes in the benign group may exhibit increased stiffness, potentially lowering specificity. Additionally, interobserver variability was not assessed, which would be important for validating the reproducibility and reliability of elastography measurements in clinical practice.

## Conclusions

In this study, multiple demographic, anatomical, and sonographic factors significantly aid in differentiating benign from malignant cervical lymph nodes. Malignancy was more common with increasing age, particularly in the 30-50 and >50 years groups, and was more frequent in female patients, whereas benign cases predominated in male patients. Lymph node location showed diagnostic relevance, with level II and IV nodes more often malignant and level V involvement usually benign. Among US features, the absence of a hilum, heterogeneous echotexture, and peripheral vascularity were strongly associated with malignancy, while a normal hilum, homogeneous echotexture, and central vascularity suggested benign pathology. Malignant nodes were significantly larger in short- and long-axis measurements, though the long-to-short-axis ratio was not a valuable differentiator. Elastography parameters showed the highest diagnostic accuracy, with malignant nodes exhibiting markedly higher mean SWE, shear wave velocity, and strain ratio than benign nodes. ROC analysis demonstrated excellent performance for both mean shear wave velocity (AUC = 0.940, cutoff = 2.195 m/s, 100% sensitivity, 85% specificity) and strain ratio (AUC = 0.956, cutoff = 1.465, 91.5% sensitivity, 99% specificity). These findings confirm that combining ultrasound morphological features, vascularity patterns, and quantitative elastography parameters offers a highly effective, noninvasive approach for distinguishing malignant from benign cervical lymphadenopathy. At the same time, histopathology remains the definitive standard for diagnosis.

## References

[1] Sakr M (2016). Cervical: lymphadenopathy. Head and Neck and Endocrine Surgery.

[2] Medeiros LJ (2009). Ioachim's Lymph Node Pathology.

[3] Sakai O, Curtin HD, Romo LV, Som PM (2000). Lymph node pathology: benign proliferative, lymphoma, and metastatic disease. Radiol Clin North Am.

[4] Azizi G, Keller JM, Mayo ML, Piper K, Puett D, Earp KM, Malchoff CD (2016). Shear wave elastography and cervical lymph nodes: predicting malignancy. Ultrasound Med Biol.

[5] Kang HJ, Seo M, Sohn YM, Yun SJ, Min SY, You MW, Yeon EK (2019). Comparison of diagnostic performance of B-mode ultrasonography and shear wave elastography in cervical lymph nodes. Ultrasound Q.

[6] Cui XW, Chang JM, Kan QC, Chiorean L, Ignee A, Dietrich CF (2015). Endoscopic ultrasound elastography: current status and future perspectives. World J Gastroenterol.

[7] Pagé G, Tardieu M, Besret L (2019). Assessing tumor mechanics by MR elastography at different strain levels. J Magn Reson Imaging.

[8] Winn N, Baldwin J, Cassar-Pullicino V, Cool P, Ockendon M, Tins B, Jaremko JL (2020). Characterization of soft tissue tumours with ultrasound, shear wave elastography and MRI. Skeletal Radiol.

[9] Youk JH, Gweon HM, Son EJ (2017). Shear-wave elastography in breast ultrasonography: the state of the art. Ultrasonography.

[10] Chiorean L, Cui XW, Klein SA (2016). Clinical value of imaging for lymph nodes evaluation with particular emphasis on ultrasonography. Z Gastroenterol.

[11] Hackett L, Aveledo R, Lam PH, Murrell GA (2020). Reliability of shear wave elastography ultrasound to assess the supraspinatus tendon: an intra and inter-rater in vivo study. Shoulder Elbow.

[12] Bhatia KS, Cho CC, Tong CS, Yuen EH, Ahuja AT (2012). Shear wave elasticity imaging of cervical lymph nodes. Ultrasound Med Biol.

[13] Albano D, Basile M, Gitto S (2024). Shear-wave elastography for the evaluation of tendinopathies: a systematic review and meta-analysis. Radiol Med.

[14] Cannella R, Agnello F, Porrello G (2025). Performance of ultrasound-guided attenuation parameter and 2D shear wave elastography in patients with metabolic dysfunction-associated steatotic liver disease. Eur Radiol.

[15] Gurun E, Ozturk M, Cakir IM, Genc AS, Bozduman O, Sazak HB, Okutan AE (2025). The efficacy of shear wave elastography in evaluating treatment response to US-guided steroid injection in patients with carpal tunnel syndrome. Acad Radiol.

[16] Kaya M, Gürün E (2022). Do deep inspiration breath-holds and free-breathing affect pancreatic tissue stiffness in shear wave elastography?. Abdom Radiol (NY).

[17] Ishibashi N, Yamagata K, Sasaki H (2012). Real-time tissue elastography for the diagnosis of lymph node metastasis in oral squamous cell carcinoma. Ultrasound Med Biol.

[18] Gürüf A, Öztürk M, Bayrak İK, Polat AV (2019). Shear wave versus strain elastography in the differentiation of benign and malignant breast lesions. Turk J Med Sci.

[19] Mattoo P, Firdose SR, Wani RA (2022). Role of acoustic radiation impulse ultrasound elastography in the assessment of cervical lymphadenopathy. NeuroQuantology.

[20] Chavoshi M, Taghavi M, Hashemi H, Davoodi M, Rouzrokh P, Aghaghazvini L (2026). Diagnostic value of shear wave elastography in differentiation between benign from malignant cervical lymph nodes. J Iran Med Counc.

[21] Gupta D, Kumar H, Bagga PK, Ohri P, Kumar H (2024). Role of ultrasound elastography in evaluation of thyroid nodules with FNAC/histopathological correlation. Int J Life Sci Biotechnol Pharma Res.

[22] Gao Y, Zhao Y, Choi S (2022). Evaluating different quantitative shear wave parameters of ultrasound elastography in the diagnosis of lymph node malignancies: a systematic review and meta-analysis. Cancers (Basel).

[23] Sasaki Y, Ogura I (2019). Shear wave elastography in differentiating between benign and malignant cervical lymph nodes in patients with oral carcinoma. Dentomaxillofac Radiol.

[24] Vineela E, Sakalecha AK, Narayanrao Suresh T (2022). Role of sonoelastography in differentiating benign from malignant cervical lymph nodes and correlating with pathology. Cureus.

[25] Gupta R, Mittal P, Harkirat TK, Kaur H, Aamir M, Malik R (2017). Ultrasound elastography for differentiating benign from malignant cervical lymphadenopathy: comparison with B-mode and color Doppler findings. J Clin Diagn Res.

[26] Moharram MA, Abd-El Maboud NM, Ahmed HA (2017). Evaluation of the role of sono-elastography in diagnosis of enlarged cervical lymph nodes. Egypt J Radiol Nucl Med.

[27] Seetharam M, Prakash MC, Pradeep HN, Nanjaraj CP, Narayana M, Ramakrishnaiah RA, Ker AR (2022). Role of sonoelastography for differentiating benign and malignant cervical lymph nodes: a cross-sectional study. J Anat Radiol Surg.

